# Protective Effects of *Tinospora crispa* Stem Extract on Renal Damage and Hemolysis during *Plasmodium berghei* Infection in Mice

**DOI:** 10.1155/2015/738608

**Published:** 2015-10-27

**Authors:** Narain Nutham, Sakuna Sakulmettatham, Suwit Klongthalay, Palatip Chutoam, Voravuth Somsak

**Affiliations:** Department of Clinical Chemistry, Faculty of Medical Technology, Western University, Kanchanaburi 71170, Thailand

## Abstract

Renal damage and hemolysis induced by malaria are associated with mortality in adult patients. It has been speculated that oxidative stress condition induced by malaria infection is involved in its pathology. Thus, we aimed to investigate the protective effects of *Tinospora crispa* stem extract on renal damage and hemolysis during *Plasmodium berghei* infection. *T. crispa* stem extract was prepared using hot water method and used for oral treatment in mice. Groups of ICR mice were infected with 1 × 10^7^ parasitized erythrocytes of *P. berghei* ANKA by intraperitoneal injection and given the extracts (500, 1000, and 2000 mg/kg) twice a day for 4 consecutive days. To assess renal damage and hemolysis, blood urea nitrogen (BUN), creatinine, and hematocrit (%Hct) levels were then evaluated, respectively. Malaria infection resulted in renal damage and hemolysis as indicated by increasing of BUN and creatinine and decreasing of %Hct, respectively. However, protective effects on renal damage and hemolysis were observed in infected mice treated with these extracts at doses of 1000 and 2000 mg/kg. In conclusion, *T. crispa* stem extract exerted protective effects on renal damage and hemolysis induced by malaria infection. This plant may work as potential source in the development of variety of herbal formulations for malarial treatment.

## 1. Introduction

Malaria is one of the world's leading killers, having a greater morbidity and mortality than any other infectious diseases, with greater mortality in children and pregnant women. Malaria is caused by a parasite of the genus* Plasmodium* and transmitted by female* Anopheles* mosquito [1]. Renal damage and hemolysis induced by malaria, especially* P. falciparum* and* P. vivax*, occur between 2 and 6% of hospitalized patients with a mortality that can reach up to 50% [2]. The pathogenesis of renal damage and hemolysis during malaria infection is multifactorial and not well characterized, but several studies suggest involvement of cytoadherence of parasitized erythrocytes and proinflammatory response as well as oxidative stress [3–5]. Moreover, parasite invasion and subsequent erythrocyte rupture also contributed to pathogenesis of hemolysis [6]. This has prompted research towards the discovery and development of new, safe, and affordable antihemolysis drugs during malaria infection. In this respect, medicinal plants are potential targets for research.

Plant extracts are commonly investigated in several parts of the world for their possible activities for using in malaria [7–10]. Moreover, many studies recently endorsed the use of traditional medicinal plants to treat diseases [11–16].* Tinospora crispa* Hook. f. and Thomson is a tree that belongs to the family Menispermaceae. This plant is widely found in Asia and Southeast Asia including Thailand and used for general weakness, malaria fevers, and chronic rheumatism [17]. It has been reported that* T. crispa* stem extract has high antioxidant property. This result is confirmed by the existence of phenolic and flavonoids such as catechin, luteolin, and borapetoside [18]. These are responsible for high antioxidant activity and other activities including anti-inflammation, antimicrobials, and anticancer [19–21]. The antioxidant properties of* T. crispa* stem extract make it useful for the treatment of diseases resulting from oxidative stress such as cardiovascular disease, cancer, liver damage, and renal disease. It also has been used as an antimalarial agent* in vivo* [22, 23]. However, it has not been reported yet to use* T. crispa* stem extract to treat renal damage and hemolysis induced malaria infection. Hence, the present study was aimed to investigate the protective effects of* T. crispa* stem extract on renal damage and hemolysis induced by* P. berghei* infection in mice.

## 2. Materials and Methods

### 2.1. Plant Material and Preparation of Crude Extract

Fresh stems of* Tinospora crispa* were collected from natural environment in Suphanburi province, Thailand, from March to April 2015. The samples were identified by Dr. Sakaewan Ounjaijean, Faculty of Pharmacy, Payap University, Chiang Mai, Thailand. Stems were minced into small pieces and dried in hot-air oven at 60°C for 72 h, after which the dried plants were ground and then extracted in distilled water at the proportion of 1 : 10 (w/v) using microwave at 360 W for 5 min [24]. Incubation at room temperature with frequent agitation was then performed for overnight. The extract was filtered through Whatman number 1 filter paper, and evaporation was then performed to remove solvent. Crude extract was kept at −20°C. Before experiment, powder extract was dissolved in distilled water to obtain appropriate doses for using in mice and volume should not be higher than 1 mL. Drug doses, expressed in mg/kg of body weight, were adjusted at the time of administration according to the weight of the mice.

### 2.2. Mice

Pathogen-free, female ICR mice, aged 4–6 weeks and weighting 25–30 g, were purchased from the National Laboratory Animal Center, Mahidol University, Bangkok, Thailand. They were kept at 22–25°C with 12 h light/dark cycle and given standard mouse pellet and water* ad libitum*. All procedures of the animal experiments were approved by the Ethical Committee on Animal Experimentation, Faculty of Medical Technology, Western University.

### 2.3. *Plasmodium berghei* Parasite

*Plasmodium berghei* strain ANKA (PbANKA) was used in this study. PbANKA was kept alive by intraperitoneal (IP) passage of 1 × 10^7^ parasitized erythrocytes in naïve mice. Parasitemia was daily monitored by microscopic examination of Giemsa stained thin blood smear. In addition, hematocrit (%Hct) was also monitored by collecting tail vein into heparinized hematocrit tubes and centrifuged at 10,000 g for 10 min. Proportion of packed erythrocytes and total blood volume was finally calculated.

### 2.4. Assessment of Renal Function Tests

Blood urea nitrogen (BUN) and creatinine were used as indicators of renal function. Mouse blood was collected from tail vein into heparinized hematocrit tubes and centrifuged at 10,000 g for 10 min. Plasma was subsequently collected and used as subjects for measurement of BUN and creatinine using an available commercial kit (BioSystems) according to manufacturer's instruction.

### 2.5. Efficacy Test* In Vivo*

Protective effect of aqueous crude extract of* T. crispa* on renal damage and hemolysis during malaria infection was done based on standard 4-day suppressive test [25]. Groups of ICR mice (5 mice of each) were randomly divided, and infection was then performed with 1 × 10^7^ parasitized erythrocytes of PbANKA by IP injection. Two hours after infection, they were given orally by gavage with 500, 1000, and 2000 mg/kg of the extracts and every 24 h twice a day for 4 consecutive days. Three control groups were used; normal controls were given either distilled water or the extract while untreated control was given only distilled water. On day 4 of experiment, tail blood was collected to assess hemolysis by measurement of %Hct. Moreover, plasma was subsequently also used to measure BUN and creatinine.

### 2.6. Statistics

Statistical analysis was done using GraphPad Prism version 5.01. The results were presented as mean ± standard error of mean (SEM). One-way ANOVA was used to compare several treatment groups. Significant differences were considered at 95% confidence,* P* < 0.05.

## 3. Results

### 3.1. Renal Damage and Hemolysis Induced by* Plasmodium berghei* ANKA Infection in Mice

**Figure 1:**
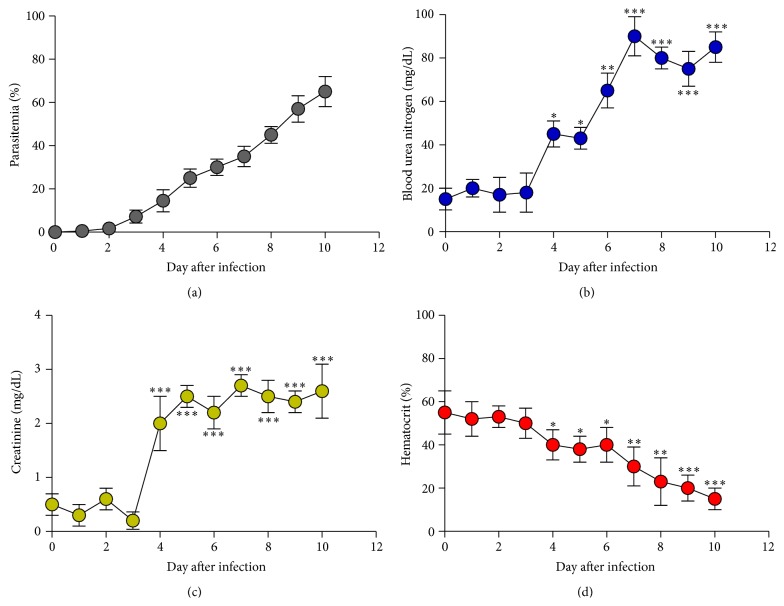
Renal damage and hemolysis induced by* Plasmodium berghei* infection. ICR mice (5 mice) were inoculated with 1 × 10^7^ parasitized erythrocytes of PbANKA by intraperitoneal injection. (a) Parasitemia, (b) BUN, (c) creatinine, and (d) hematocrit levels were daily monitored. Results were expressed as mean ± SEM. ^*∗*^ *P* < 0.05, ^*∗∗*^ *P* < 0.01, and ^*∗∗∗*^ *P* < 0.001, compared to day 0 after infection.

Parasitemia was first detectable on day 1 after infection with a parasitemia of 0.5% and reached 65% on day 10 (Figure 1(a)). Next, we observed that BUN and creatinine levels were markedly increased in infected mice, and first significant (*P* < 0.05 and* P* < 0.001) increases were found on day 4 after infection (Figures 1(b) and 1(c)). Additionally, we also observed progressive decreases of %Hct in infected mice in response to the presence of parasitemia (Figure 1(d)).

### 3.2. Protective Effect of* Tinospora crispa* Stem Extract on Renal Damage during* Plasmodium berghei* ANKA Infection in Mice

**Figure 2:**
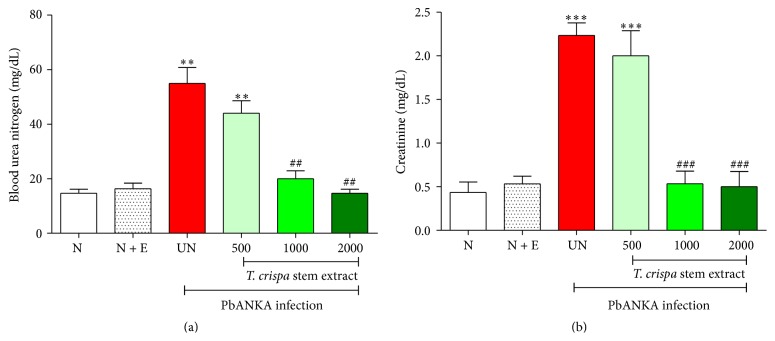
Protective effect of* Tinospora crispa* stem extract on renal damage induced by* Plasmodium berghei* infection. ICR mice (5 mice of each) were inoculated with 1 × 10^7^ parasitized erythrocytes of PbANKA by intraperitoneal injection. The extracts (500, 1000, and 2000 mg/kg) were given orally by gavage for 4 consecutive days. On day 4 of experiment, (a) BUN and (b) creatinine levels were then measured. Results were expressed as mean ± SEM. ^*∗∗*^ *P* < 0.01 and ^*∗∗∗*^ *P* < 0.001, compared to normal control, and ^##^ *P* < 0.001 and ^###^ *P* < 0.001, compared to untreated control. N: normal control, N + E: normal mice treated with 2000 mg/kg of extract, and UN: untreated control. The results came from 3 independent experiments.

It was found that* T. crispa* stem extract showed protective effect on renal damage induced by PbANKA infection in mice, and the highest activity was found at dose of 2000 mg/kg of extract treated group (Figure 2). The BUN and creatinine levels were increased significantly (*P* < 0.01 and* P* < 0.001, resp.) by malaria infection (untreated group) and infected mice treated with 500 mg/kg of the extract, compared to normal control. Interestingly, after oral administration of* T. crispa* stem extract, BUN and creatinine levels declined significantly (*P* < 0.01 and* P* < 0.001, resp.), especially at doses of 1000 and 2000 mg/kg, compared to untreated control. No effect was observed in normal mice treated with this extract. However,* T. crispa* stem extract could not be able to decrease parasitemia levels.

### 3.3. Antihemolytic Effect of* Tinospora crispa* Stem Extract during* Plasmodium berghei* ANKA Infection in Mice

**Figure 3:**
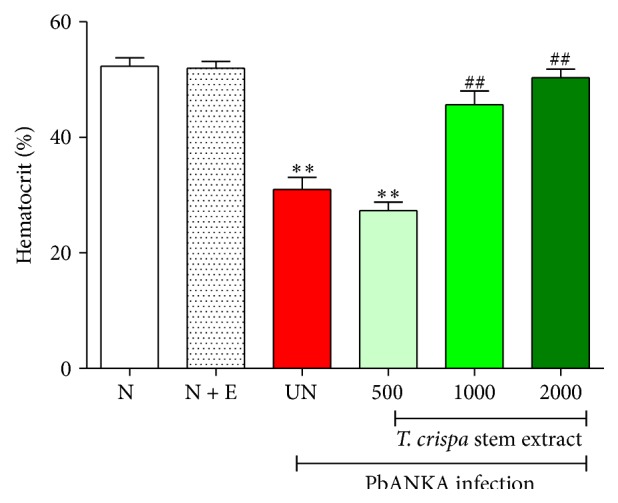
Protective effect of* Tinospora crispa* stem extract on hemolysis induced by* Plasmodium berghei* infection. ICR mice (5 mice of each) were inoculated with 1 × 10^7^ parasitized erythrocytes of PbANKA by intraperitoneal injection. The extracts (500, 1000, and 2000 mg/kg) were given orally by gavage for 4 consecutive days. On day 4 of experiment, hematocrit levels were then measured. Results were expressed as mean ± SEM. ^*∗∗*^ *P* < 0.01, compared to normal control, and ^##^ *P* < 0.001, compared to untreated control. N: normal control, N + E: normal mice treated with 2000 mg/kg of extract, and UN: untreated control. The results came from 3 independent experiments.

*T. crispa* stem extract exerted antihemolytic effect against PbANKA infected mice. As shown in Figure 3, hemolysis was observed in untreated control and infected mice treated with 500 mg/kg of the extract as indicated by decreasing of %Hct with significance (*P* < 0.01), compared to normal control. Interestingly, %Hct was significantly (*P* < 0.01) increased in infected mice treated with 1000 and 2000 mg/kg of the extract, compared to untreated control. The highest effect was found at dose of 2000 mg/kg of the extract. No effect was observed in normal mice treated with this extract. Moreover, prolonged survival time of infected mice treated with this extract was also observed.

## 4. Discussion

In the current study, we provide evidence that describes the protective effect of* T. crispa* stem extract on renal damage and hemolysis induced by* P. berghei* infection in mice. The onset of renal damage and hemolysis in ICR mice infected with PbANKA come out from day 4 after infection and the incidence of renal damage and hemolysis were confirmed through manifestations such as increasing of BUN and creatinine as well as decreasing of %Hct in infected mice. Renal damage and hemolysis are proposed to be a consequence of parasite adhesion and exacerbated immune response against products of oxidative stress released during infection [26]. In addition, parasitized erythrocyte sequestration at the microvascular site of renal has also been discussed to induce renal damage. The destruction of erythrocytes during blood stage infection accumulates high levels of toxic-free heme that has ability to induce oxidative stress from production of hydroxyl radicals via Fenton/Haber-Weiss reaction [27]. Moreover, heme-derived oxidative stress is considered to be a main factor in the iron-induced lipid peroxidation resulting in the formation of oxidized LDL [28]. Therefore, these conditions derived by blood stage of infection result in renal damage and hemolysis.

However, protective effects of renal damage and hemolysis induced by malaria infection were found in infected mice treated with* T. crispa* stem extract. Polyphenols and flavonoids show high antioxidant and anti-inflammation properties and then protect oxidative stress development [17].* T. crispa* stem extract has been shown to contain high amounts of polyphenols and flavonoids [29]. Thus, the potent antioxidant activity of this extract might play a central role in protecting renal damage and hemolysis from oxidative stress induced by malaria infection. Moreover,* T. crispa* stem extract has been reported to have antimalarial activity against* P. yoelii* infection in mice [22]. So, antimalarial activity to inhibit* P. berghei* growth and subsequently inhibit renal damage and hemolysis might also be mentioned.

## 5. Conclusions

Taken together, our results suggest that* T. crispa* stem extract shows protective effects against* P. berghei*-induced renal damage and hemolysis. Appropriate pharmaceutical strategies might now be devised to increase the low bioavailability of this extract and to protect against rapid* in vivo* metabolic transformation, in such a way to make them more amenable as alternative antimalarial drug in combination therapies.
